# Applications of NMR in Dairy Research

**DOI:** 10.3390/metabo4010131

**Published:** 2014-03-04

**Authors:** Anthony D. Maher, Simone J. Rochfort

**Affiliations:** 1 Biosciences Research Division, Department of Environment and Primary Industries, 5 Ring Rd Bundoora, Victoria 3083, Australia; E-Mail: anthony.maher@gmail.com; 2 School of Systems Biology, LaTrobe University, Bundoora, Victoria 3083, Australia

**Keywords:** nuclear magnetic resonance, metabolomics, bovine, rumen, milk, review

## Abstract

NMR is a robust analytical technique that has been employed to investigate the properties of many substances of agricultural relevance. NMR was first used to investigate the properties of milk in the 1950s and has since been employed in a wide range of studies; including properties analysis of specific milk proteins to metabolomics techniques used to monitor the health of dairy cows. In this brief review, we highlight the different uses of NMR in the dairy industry.

## 1. Inroduction

### Early Nuclear Magnetic Resonance Studies on Milk and Milk Products

Milk is a complex, multi-phasic colloidal suspension that contains complete food for infants and is produced on an industrial scale for human consumption. The application of NMR spectroscopy to analyse milk dates as far back as the 1950s, when Odeblad and Westin were able to identify three distinct “components” in the ^1^H NMR resonance signal (at 21 MHz) from human milk, which they labelled *W*, *T* and *F*. By measuring relative resonance frequencies and spin-spin and spin-lattice relaxation times, they were able to assign components *W* and *T* to the milk plasma, with *W* identified as being the resonance from water, while the *T* component was a shoulder peak 0.5 ppm downfield of this (possibly from the lactose in the sample). The *F* component was identified as coming from the milk fat. They measured spin-lattice relaxation times for milk plasma from several samples collected in the days after birth, but found no significant trend [1]. In 1972, Chandan *et al.* used ^1^H NMR to investigate the structural organization of the milk fat globule membrane (MFGM). By measuring the 60 MHz ^1^H NMR spectrum of preparations of MFGM in various solvents, they were able to identify characteristic resonance patterns from lipids, including the assignment of different protons within lipid chains (e.g., CH_3_, CH_2_ and CH=CH protons, *etc.*) and contrast these with those resonance patterns from denatured proteins (which gave rise to amino acid signals) to establish that the MFGM is structurally and functionally different from the plasma membrane [2]. Although it would be many years before the term “metabolomics” was coined, these studies illustrate that from shortly after its discovery in the 1940s, scientists were already keen to harness the analytical power of NMR to measure chemical and physical properties for the extraction of biological information from sets of samples. As the sensitivity and resolution of NMR spectrometers increases and multivariate statistical techniques become more sophisticated, the depth and breadth of the questions that can be answered using NMR metabolomics in research has greatly expanded.

In this paper, we review the extent to which NMR has been applied in research relevant to the dairy industry, from NMR studies on the physio-chemical properties of milk and other dairy products, to the more recent metabolomic analysis of these samples.

## 2. ^1^H NMR Studies

The vast majority of NMR-based metabolomics studies have used biological fluids as the sample of choice. This is because these are relatively easy to collect; sample preparation for NMR is generally straightforward, and they provide a wealth of biochemical information that is sensitive to the dynamic metabolic status of the organism they have been collected from. In the dairy industry, milk is routinely collected from lactating cows and can provide researchers not only with information about the animal providing the sample, but also with the potential for that sample to be used in industry, such as for making cheese and other products.

In 1999, Belloque *et al.* published a review of the application of NMR techniques in dairy. The described NMR studies ranged from investigations focused on specific molecules (e.g., proteins, such as caseins; free or protein-bound carbohydrates) to studying the physical properties of milk (e.g., lipid behaviour under different thermal conditions; water interactions in gels or liquids as relevant for processes, such as renneting) [3].

Since then, NMR has continued to be used as a tool in dairy science. Low-field or time domain NMR (TD-NMR), a low-cost alternative to high-field NMR, has been used to investigate the freezing properties of ice cream with different fats incorporated, to investigate the texture attributes of milk drinks, to characterize fat and water in cheese, to investigate the solid fat content in anhydrous milk fat blends and, in conjunction with high field NMR, the properties of mozzarella di bufala Campana cheese [4,5,6,7,8]. A review in 2007 highlighted the benefits of utilising 2D NMR experiments compared to 1D protocols for the provision of rapid diffusion measurements in low-field NMR studies of dairy products (and some other food stuffs) [9]. Recently, a method for the rapid (less than two minutes) measurement of fat and moisture in cheese by TD-NMR has been developed. A calibration sample set was used to measure fat and moisture by standard methods, and spin-lattice relaxation (T_1_) and spin-spin relaxation (T_2_) data were acquired, as well. Using partial least squares (PLS) regression techniques, this calibration set was used to generate prediction equations (with an error of less than 1%) that were then used for determining the levels of fat and moisture in the cheeses that had only been assessed by the TD-NMR method, saving considerable time and expense [10]. Low-field NMR has also been used to investigate the stability of model milk powders. Microbial activity, lysine content, water activity and glass transition properties are key factors in retaining value in milk powders. Schmitz-Schug *et al.* demonstrated that low field 1H NMR and differential scanning calorimetry both suggest that lactose crystallizes into different crystal forms and that analysis of the molecular mobility together with this physical state information could be used to optimize dairy formulations with respect to chemical stability [11].

Milk can be analysed successfully by NMR without pre-treatment. Hu *et al.* established that whole milk could be analysed by NMR, although the addition of 10% D_2_O did allow for easier locking and increased resolution [12]. These authors were also able to obtain excellent ^13^C and ^1^H−^13^C HSQC (Heteronuclear Single Quantum Coherence) spectra on whole milk and assigned milk metabolites (including lactose, glycerol and lipids). Interestingly, they noted that there was a significant difference in the signal-to-noise and resolution between homogenized milk and non-homogenised milk; suggesting that in the latter, the broad resonances were due to very large milk fat globules that were broken down to smaller globules in the homogenized milk [12]. This result suggests that NMR may be a useful tool for the assessment of milk fat globule size. Milk lipids have been characterized by NMR. Although ^1^H NMR is more sensitive and, therefore, data acquisition more rapid, for the identification and quantification of lipids, ^13^C NMR is more informative. With the use of commercially available standards, Andreotti *et al.* were able to identify 13 different acyl groups in triacylglycerols (TAGs) and compared buffalo milk TAGs to cow milk TAGs. The major difference was in C_10:0 _content, with buffalo milks having almost twice as much capric acyl content compared to the cow milks [13]. In a comprehensive study of cheese lipids by 1H and 13C NMR techniques, the lipolytic and lipid oxidation processes in pecorino sardo cheese were studied, demonstrating the use of these techniques for the study of lipids with relevance to flavour and human health [14]. To identify the minor metabolites in milk, Hu *et al.* defatted (by centrifugation), removed protein (by precipitation) and freeze dried milk to enable the acquisition of both ^1^H−^15^N HMBC and ^1^H−^13^C HMBC NMR data. Using these protocols, the authors were able to assign resonances, due to creatine, citrate, lecithin and N-acetyl carbohydrates; suggesting that NMR could be a useful tool in the quantitation of these molecules [12]. NMR continues to be used for the investigation of physic-chemical processes, e.g., the diffusion of casein aggregates and casein behaviour in the renneting process [15,16]. The latter study suggested that there are two types of casein that have different diffusion properties in the milk and that not all the casein molecules aggregate in renneting [16]. Diffusion NMR studies coupled with results from circular dichroism show that the major whey protein, β-lactoglobulin, interacts with certain phospholipids in a hydrophilic manner and that this can affect the tertiary structure and, by implication (although further study is warranted), the function of these proteins during heat treatment, common in milk processing [17]. NMR has been used to discriminate between Sardinian sheep milk cheese made from HT (heat treated) milk and raw milk utilising MRI techniques that allowed differentiation based on the interaction of water protons with proteins [18]. The method was relatively rapid (6–18 min) and, beyond product differentiation, suggests that the technique may be of use in studying, at a more fundamental level, the processes involved in cheese making. The formation of dairy gels (e.g., yoghurt) has been investigated by NMR, and an increase in lactate was observed in real time as the microbes fermented the milk, suggesting that the technique may prove useful in the evaluation of new bacterial strains for dairy products [19]. These authors have also demonstrated the utility of NMR for monitoring the changes in liquid fat content in dairy gels, again demonstrating the utility of NMR in understanding complex processes in food processing and production [20].

Metabolomics, a relatively new area of study, is the high-throughput identification and quantification of metabolites in a particular sample. NMR is one of the major analytical tools for metabolomics and particularly amenable for the study of biofluids, including milk, as recently reviewed [21]. A variety of questions have been addressed using NMR metabolomics techniques. The techniques have been applied to assess the relationships between milk metabolic profiles and the technological properties of milk. Sundekilde *et al.* used principal components analysis (PCA) models to show that the ^1^H NMR measured metabolomes of milk formed separable clusters on breed (Holstein *vs*. Jersey) and coagulation properties. Although not done in this study, it would be highly informative if supervised analyses, such as partial least squares discriminant analysis (PLS-DA), were performed, since this could determine whether these technological properties can be robustly predicted from these data [22]. In another study, ^1^H NMR metabolomics was combined with artificial neural network (ANN) analysis to quantify the relative amounts of goat’s and cow’s milk in synthetic mixtures [23].

NMR-metabolomics coupled with isotope ratios mass spectroscopy (IRMS) allowed Sacco *et al.* to discriminate between milk samples from Southern Italy and milks obtained from Central-Eastern Europe. However, since the milks from the Central-Eastern Europe were obtained from commercial outlets and had been processed (e.g., pasteurized), while the other milks were obtained directly from Italian farms and were raw, the data analysis and conclusions are less straightforward. The authors did observe a greater spread in data from the raw milks, while the data from the processed milks clustered quite closely, and they suggested that this was likely due to the processing; however, without further study, this remains uncertain. Of most interest is that the NMR/IRMS-based data allowed this separation of the samples, whereas data from high performance ion chromatography (HPIC) and inductively coupled plasma atomic emission spectroscopy (ICP-AES) did not, highlighting the value of the information-rich NMR technique [24].

More recently, ^1^H NMR of milk samples has been used to monitor metabolic variation over lactation stages in dairy cows. Klein *et al.* demonstrated that markers of energy metabolism status, such as citrate, acetone and β-hydroxybutyrate, were elevated during early lactation, then decreasing over the first 70 days, before stabilizing, indicating energy imbalance in these cows as lactation was initiated. Decreases were also observed for phosphocholine and glycine over the same period [25]. These authors also performed a more detailed analysis of milk from 264 Holstein-Friesian animals, and identified the glycerophosphocholine to phosphocholine ratio as a marker for risk of ketosis [26]. Such studies suggest the potential of NMR in monitoring animal health and metabolic status. Ilves *et al.* used a combined NMR and mass spectrometry approach to identify systematic metabolic changes over lactation in both blood and milk of cows, but found little correlation between the two biofluids using separate PCA models [27]. It may be the case that more in-depth statistical analyses may assist in pulling out subtle correlations between these biofluids, such as statistical heterospectroscopy (SHY) [28,29]. Metabolomics analysis of the urinary metabolite profile of dairy cows demonstrated a link between nitrogen intake (cows were fed diets with four different levels of crude protein), as well as nitrogen efficiency by PCA and PLS regression modelling. The authors found that although nitrogen efficiency could be predicted reasonably well (R = 0.74), it was not possible to identify a single metabolite that could be used as a marker to predict nitrogen efficiency [30]. This is not necessarily surprising and demonstrates the benefits of a multivariate metabolomics approach over reliance on a single biomarker. NMR metabolomic approaches have also been employed for the validation of nutritional information. Monakhova *et al.* demonstrated that SIMCA (soft independent modelling of class analogy) statistical modelling could differentiate between lactose-containing milk, lactose-free milk, as well as oat, soy and rice milk substitutes. The authors also demonstrated they could use NMR to quantitate the amount of lactose present, although they found it necessary to remove fat, due to signal broadening [31]. NMR milk metabolomics coupled with genome-wide association studies (GWAS) has allowed the heritability of 31 milk metabolites to be estimated. The heritability of some molecules (lactic acid, isobutyrate, acetic acid, fumaric acid and galactose) were low (less than 0.11), but several were high, with orotic acid and β-hydroxybutyrate demonstrating heritability of 0.86 and 0.87, respectively [32]. This data suggests that it would be possible to breed animals that produced milk with more or less of these highly heritable molecules, which may have advantages in processing or human health.

Several studies have applied ^1^H NMR metabolomics to cheese samples. ^1^H NMR spectra acquired over the course of ripening demonstrated systematic changes over time, with lactose at Day 0 being almost completely converted to lactate and ketone bodies by Day 60. Furthermore, orthogonal partial least squares (OPLS) analyses suggested that the spectral data could predict the bacterial strain supplement (when comparing *B. lactis* *vs*. *L. casei*), indicating the influence these added species can have on the metabolic profiles of these products [33]. Lamanna *et al.* combined high resolution magic angle spinning (HR-MAS) with liquid NMR of aqueous extracts to monitor the degradation of samples of Italian soft cheeses, showing relative increases in signals assigned to glucose (although it is likely that lactose contributed to these signals, since many of the lactose signals are co-resonant with those of glucose), lactate, phosphocholine, glycerophosphocholine and creatine [34]. In 2007, Gianferri *et al.* used ^1^H NMR to measure the metabolic profile of buffalo mozzarella, which may have applications for assessing the authenticity of such products [6]. Along a similar vein, a more recent study (2012) demonstrated that high resolution magic angle spinning solid-state NMR techniques could be used to discriminate the region of origin of buffalo mozzarella cheese [35]. GC-MS (gas chromatography–mass spectrometry) and ^1^H NMR together identified cyclopropyl and ω-cyclohexyl fatty acids of microbial origin in 200 milk samples from dairy cows fed with different forages. The authors studied silage, milk and cheese, discovering that cyclopropyl fatty acids (about 0.1% of milk fat) were only in milk samples from cows fed with forages containing maize silage and that these lipids could also be detected in cheese made from this milk. This is a significant finding, since milk from cows fed maize silage cannot be used in making cheeses, such as Parmigiano-Reggiano [36]. This study demonstrates the utility of metabolomics approaches for following the effect of animal nutrition on the end product, as well as provenance determination. NMR metabolomics techniques have been demonstrated in determining not only the provenance of cheeses [37,38], but also used to investigate metabolite changes during cheese ripening [39,40].

Beyond milk and milk products, several studies have applied ^1^H NMR to samples, such as blood or other tissues, to understand bovine metabolic responses to various pathophysiological stimuli. Bertram *et al.* were able to obtain arterial, portal and hepatic blood plasma to investigate splanchnic metabolism in response to the infusion of short-chain fatty acids (SCFA). Using PCA and PLS-DA for multivariate analyses, there were clear increases in β-hydroxybutyrate, acetate and propionic acid following the infusion of SCFA [41]. This type of study demonstrated that there were distinct differences between the different types of blood, depending on where from the animal it was collected. This has implications for studies of bovine metabolism in which blood has been collected from the tail vein. It is important to note that blood collected from the tail may not reflect metabolic activity closer to the gut (which is reflected in the portal blood) and may obscure the influence of metabolic activity in the rumen. In more recent investigations, this group has used ^1^H NMR as a non-selective profiling tool for exploring propylene glycol (PG) toxicity and to assess the effects of different diets on the metabolism of rumen epithelial tissue [42,43]. PG toxicity resulted in blood plasma metabolic profiles that were distinct from those from cows that did not suffer toxicity. However, since the number of samples was low (N = 3), further studies will confirm whether this condition can be predicted from NMR spectra. More recently, milk has been analysed to determine if there are correlations between somatic cell count (SCC) and milk metabolome. In an analysis of 876 samples, several biomarkers associated with SCC were identified, including lactate, acetate, hippurate, isoleucine, butyrate and fumarate [44]. Amatej *et al.* have employed NMR metabolomics to investigate the impact of diet on the bovine rumen metabolome [45]. The authors examined rumen fluid collected via cannula using a tube fitted with a strainer and a syringe from 60 rumen-fistulated cows at five different time points during the feeding study. Not only were the authors able to discriminate between samples from animals with different amounts of cereal grain in their diet, they were able to see specific metabolite changes (xanthine, uracil, alanine and endotoxin) that suggested an unhealthy change in rumen microbiome. It would have been even more interesting if the authors had been able to examine blood or milk from the same animals to see if any indicators of rumen acidosis could be detected in these more readily available biofluids. Sun *et al.* were able to demonstrate the utility of ^1^H NMR for the detection of ketosis. In an analysis of plasma from 81 Holstein divided into three subsets (cows with clinical ketosis, cows with subclinical ketosis and healthy control cows), it was demonstrated that both clinical ketosis and subclinical ketosis could be predicted from OPLSDA (orthogonal partial least squares discriminant analysis) of the data [46].

In an attempt to provide a catalogue of bovine metabolites, including those of microbial origin and found in the rumen, the developers of the Human Metabolome Data Base (HMDB) have recently released an analogous reference source for beef and dairy cows, which includes both NMR and MS data [47,48]. This database allows for searching information about metabolites found in beef and dairy cattle and includes both literature and experimentally-derived information on bovine meat, serum, milk, urine and ruminal fluid, providing agricultural researchers with a valuable resource.

## 3. ^31^P NMR Studies

Some studies have explored the properties of the ^31^P spectrum, given the abundance of phosphorylated compounds in milk products. In 1985, Belton *et al.* observed three peaks in the ^31^P spectrum of skim milk and assigned these to inorganic phosphate (Pi), the seryl phosphate (SerP) residues of casein and glycerophosphocholine (GPC) [49]. ^31^P NMR was also used to quantitatively characterize the phospholipids from the MFGM after developing a monophasic mixture preparation [50]. 2D MAS ^31^P NMR has been used to investigate the effect of polyphosphate salts, an important food additive for the dairy food industry, in milk and processed cheese before and after heat treatment. ^31^P NMR proved useful in assessing the degree of chelation with calcium and had a measurable effect on casein mobility in the matrix [51]. This suggests that physico-chemical properties of mixtures important in dairy food processing can be studied further by these NMR techniques. The quality attributes of milk have also been studied by ^31^P NMR, with changes in phosphoglyceride composition in UHT (ultra-high temperature) milk followed by NMR for three different storage temperatures. The authors suggested that changes in phosphoglycerides could be the consequence of a phosphodiesterase enzyme of bacterial origin, able to survive UHT processing; a conclusion supported by observations made when the inoculation of sterile milk with *Pseudomonas fluorescens* led to similar changes in milk composition as in the UHT milks studied [52].

^31^P NMR has also been used to investigate the differences in phosphorous-containing molecules between buffalo milk, milk ultrafiltrate and milk fat. For comparison, corresponding data for cow milk samples were obtained, with identification of resonances correlating to glycerophosphorylcholine, glycerophosphorylethanolamine, inorganic orthophosphate, serylphosphate residues of casein, phosphocreatine, N-acetylglucosamine-1-phosphate, glucose-6-phosphate, galactose-1-phosphate, phosphorylethanolamine, phosphorylcholine, glycerol-1-phosphate, phosphatidylcholine, phosphatidylethanolamine, phosphatidylserine, sphingomyelin, and phosphatidylinositol [53]. Although the authors concluded that there was little difference between cow and buffalo milk with respect to the identity of small phosphorous-containing molecules, it would have been interesting to extend this study to include a larger set of animals (more than two of each) and determine if there was variation in the quantities of these molecules.

The use of ^31^P NMR techniques (along with alternative methodologies) for the analysis of milk lipids has been discussed in a recent review and was recognized as a significant technique by the authors, although one that requires “more detailed studies to be accurately applied for the determination of PL (phospholipids) composition of dairy products” [54]. As indicated by the studies described here, there is great potential for the ^31^P technique to be used as an analytical platform for metabolomic analyses, as is now becoming routine for ^1^H NMR spectral data.

## 4. Conclusion

Improvements in both analytical methodologies and data handling have led to an increased interest in applications, such as metabolomics, in the dairy industry. Although this review has focused on NMR, other techniques, such as GC-MS, LCMS (liquid chromatography mass spectrometry), NIR and FTIR, (Fourier transform infrared spectroscopy) have been used extensively in dairy related studies. Indeed, a recent review in this area led the authors to suggest that coupling the data from these multiple platforms via chemometric techniques may lead to greater insights than possible via a single technique [55]. The utility of NMR for dairy research has been demonstrated in many and diverse applications; from studies centring on the analysis of the physico-chemical properties of milk and milk products, through metabolomics analysis linking milk and blood metabolic signatures, to metabolome-genome-wide association analyses to understand the heritability of certain milk factors. Whether employed in conjunction with other analytical techniques or not, it seems certain that NMR will continue to be used in the dairy industry and bring new insights for product stability and development, provenance determination and animal health and wellbeing.
